# The association between need frustration and subjective well-being among Chinese vocational college students in physical education contexts: a moderated mediation model

**DOI:** 10.3389/fpsyg.2026.1794435

**Published:** 2026-07-21

**Authors:** Wenpei Sha, Jian Zhang, Wenhai An, Ku Faridah Ku Ibrahim, Zhiwen Zhang

**Affiliations:** 1 School of Agriculture and Engineering, Nanyang Vocational College of Agriculture, Nanyang, Henan, China; 2 UCSI University, Kuala Lumpur, Malaysia; 3 Mental Health Education Center, Nanyang Vocational College of Agriculture, Nanyang, Henan, China; 4 Faculty of Sports and Health, Chongqing Electronic Information College, Chongqing, China

**Keywords:** cognitive reappraisal, moderated mediation, psychological need frustration, sport focus disruption, subjective well-being, vocational students

## Abstract

**Introduction:**

This study examined associations between psychological need frustration and subjective well-being among vocational college students in physical education contexts, with a focus on cognitive reappraisal and sport-related attentional disruption.

**Methods:**

Drawing on Self-Determination Theory, emotion regulation theory, and attentional control perspectives, we tested a moderated mediation model in which cognitive reappraisal statistically accounted for part of the association between psychological need frustration and subjective well-being, and sport focus disruption moderated the association between psychological need frustration and cognitive reappraisal. Data were collected from 2,344 vocational college students in China (*M* = 19.2 years, *SD* = 1.3; 52% female) using standardized questionnaires. PROCESS Model 7 with 5,000 bootstrap samples was used to examine conditional associations, controlling for age, gender, and weekly exercise time.

**Results:**

Psychological need frustration was negatively associated with subjective well-being, whereas cognitive reappraisal was positively associated with subjective well-being. Sport focus disruption moderated the association between psychological need frustration and cognitive reappraisal: this association was not significant at low levels of sport focus disruption but was positive and stronger at average and high levels. The conditional indirect association between psychological need frustration and subjective well-being through cognitive reappraisal was significant only at average and high levels of sport focus disruption.

**Discussion:**

These findings suggest a conditional pattern in which cognitive reappraisal may reflect a compensatory regulatory process under higher attentional disruption. Given the cross-sectional design, all findings should be interpreted as associations rather than evidence of causal or temporal mediation.

## Introduction

Subjective well-being (SWB) has become a central concern in educational psychology, as it is closely associated with students’ psychological adjustment and developmental outcomes (Diener, 1984). Recent studies further show that well-being and anxiety are shaped by contextual conditions across levels, including macroeconomic stability, urbanization, and classroom learning structures (Geng and Hassan, 2025; Marques et al., 2025). Vocational college students have attracted increasing attention due to their heightened vulnerability to mental health challenges compared with students in traditional academic universities (Solberg et al., 2023). In China, vocational education has expanded rapidly, now enrolling over 30 million students nationwide (China Education Online, 2025). Within this context, vocational education is often perceived as a less prestigious pathway, which may further shape students’ self-evaluations and motivational experiences. Accumulating evidence indicates that vocational college students experience considerable psychological strain, with prevalence rates of mental health symptoms frequently exceeding 30% (Huang et al., 2019; Zhou et al., 2024). Understanding the psychological factors associated with their subjective well-being is therefore of both theoretical and practical importance.

Vocational college students in physical education settings represent a context in which basic psychological needs, emotion regulation demands, and performance-related pressures are simultaneously salient and ecologically intertwined. Three features make this population particularly informative for the present study. First, vocational students face multiple stressors that can activate psychological need frustration, including career transition pressure, the perceived lower prestige of vocational education, and employment uncertainty. Second, physical education courses involve recurring performance evaluation, skill-based assessment, and peer comparison—features that directly engage perceptions of competence and autonomy. Third, the co-occurrence of these educational and sport-related pressures means that both need frustration and emotion regulation demands are likely to be salient in students’ daily course participation. Unlike general college samples, where such pressures may be diffuse or intermittent, vocational physical education students encounter a concentrated intersection of need-relevant stressors and regulatory challenges, providing a theoretically informative and ecologically meaningful setting for examining the proposed conditional associations.

Self-Determination Theory (SDT) provides a useful framework for examining variations in well-being across educational contexts (Ryan and Deci, 2000). According to SDT, the satisfaction of three basic psychological needs-autonomy, competence, and relatedness-is essential for optimal functioning, whereas psychological need frustration reflects experiences that actively thwart these needs and has been statistically associated with maladaptive emotional and motivational outcomes (Vansteenkiste and Ryan, 2013; Collie et al., 2019). Research has consistently shown that need frustration is statistically associated with diminished well-being in student populations (Reed-Fitzke and Lucier-Greer, 2021). Vocational education settings, which often involve frequent evaluation, skill-based assessment, and social comparison, may amplify perceptions of competence threat and external pressure, potentially intensifying experiences of psychological need frustration.

Emotion regulation may help explain the association between psychological need frustration and subjective well-being. Cognitive reappraisal—defined as the reinterpretation of emotionally evocative situations—is widely regarded as an adaptive regulation strategy and has been associated with better emotional adjustment and higher well-being (Gross, 2002; Gross and John, 2003; Balzarotti et al., 2016). Broader evidence also indicates that emotional intelligence and social skills are closely connected with anxiety, highlighting the relevance of emotion-related competencies for students’ psychological adjustment (Ramos-Galarza et al., 2024). Within the process model of emotion regulation (Gross, 1998; Gross, 2015), cognitive reappraisal is classified as an antecedent-focused strategy that operates early in the emotion-generative process, distinguishing it from response-focused strategies such as expressive suppression and attentional deployment strategies such as distraction. Cognitive reappraisal also involves cognitive control processes that support the reinterpretation of emotionally salient information (Ochsner and Gross, 2005). Because psychological need frustration challenges students’ self-related appraisals and meaning-making processes, cognitive reappraisal is a theoretically relevant strategy for managing need-frustrating experiences.

However, the association between psychological need frustration and cognitive reappraisal may not be uniformly negative. In some contexts, need-frustrating experiences may be linked to reduced regulatory capacity; in others, they may increase students’ perceived need to reinterpret stressful or self-threatening situations. From a compensatory regulation perspective, students who experience need frustration may report greater use of cognitive reappraisal as an effortful attempt to make sense of pressure, competence threat, or social difficulty. Thus, cognitive reappraisal may function not only as a marker of regulatory capacity, but also as a coping-oriented response that becomes more salient when students encounter psychologically demanding experiences.

Sport focus disruption may further shape this association. Sport focus disruption refers to difficulties in maintaining task-relevant attention during performance situations (Smith et al., 2006). Drawing on Attentional Control Theory (Eysenck et al., 2007) and Self-Regulation Theory (Baumeister and Vohs, 2007), performance-related attentional disruption can be understood as a condition under which regulatory demands become especially salient. In physical education settings, students who experience sport focus disruption may be more aware of performance pressure, competence concerns, and self-evaluative thoughts. Under such conditions, psychological need frustration may be more strongly associated with reported cognitive reappraisal, because students may attempt to reinterpret need-frustrating experiences to maintain psychological adjustment. From this perspective, sport focus disruption is not merely an additional source of strain, but a boundary condition that may amplify the association between need frustration and compensatory cognitive reappraisal.

**Figure 1:**
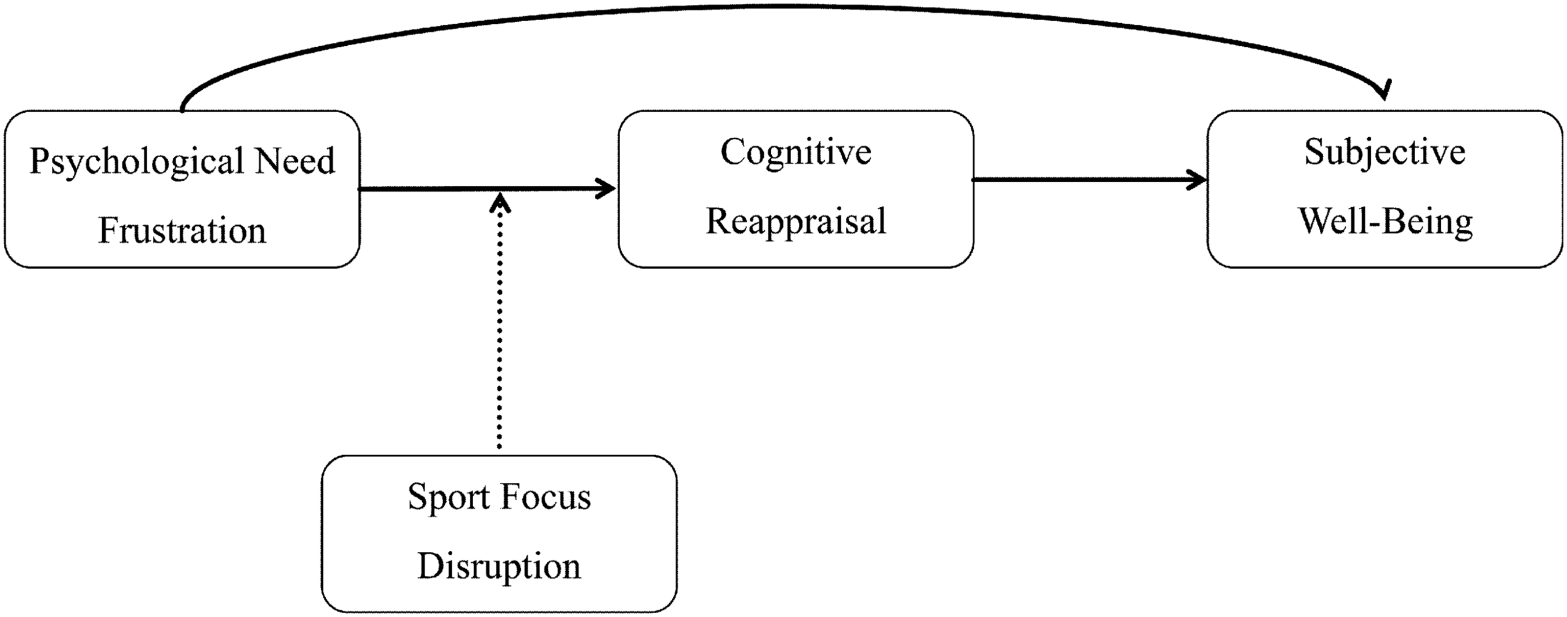
Hypothesized moderated mediation model.

The present study examines a moderated mediation model in which psychological need frustration is linked to subjective well-being through cognitive reappraisal, with sport focus disruption examined as a moderator of the need frustration–reappraisal association (Figure 1). By situating this model in vocational physical education settings, the study aims to clarify how need-related strain, emotion regulation, and sport-related attentional disruption are jointly associated with students’ subjective well-being.

### Hypotheses development

Building on the theoretical foundations outlined above, we develop specific hypotheses grounded in Self-Determination Theory, the process model of emotion regulation, Attentional Control Theory, and Self-Regulation Theory.

#### Need frustration and subjective well-being

Self-Determination Theory (SDT) posits that the fulfillment of three basic psychological needs—autonomy, competence, and relatedness—is essential for psychological growth and well-being (Ryan and Deci, 2000; Ryan and Deci, 2017). While need satisfaction promotes optimal functioning, need frustration reflects experiences that actively thwart these needs and has been associated with maladaptive emotional and motivational outcomes (Vansteenkiste and Ryan, 2013). Importantly, need frustration represents a distinct construct rather than merely low need satisfaction, as it captures experiences of pressure, incompetence, and rejection (Bartholomew et al., 2011). Accumulating evidence indicates that psychological need frustration is statistically associated with diminished well-being in educational contexts. For example, Collie et al. (2019) reported that need frustration was positively associated with emotional exhaustion and negatively associated with academic well-being. Similarly, Reed-Fitzke and Lucier-Greer (2021) found that autonomy and relatedness frustration were associated with lower life satisfaction. Prior research has also linked relatedness frustration in school contexts with poorer affective well-being (Schmidt et al., 2020). In sport-related contexts, need frustration has also been statistically associated with heightened psychological strain and maladaptive affective outcomes (Vansteenkiste and Ryan, 2013). Such contextual features may intensify experiences of psychological need frustration and, in turn, relate to diminished subjective well-being.

*H1*: Psychological need frustration is negatively associated with subjective well-being.

#### The mediating role of cognitive reappraisal

Emotion regulation theory emphasizes how individuals manage emotional experiences in shaping psychological well-being (Gross, 2002; Gross, 2015). Cognitive reappraisal, defined as the reinterpretation of emotionally relevant situations to alter their emotional impact, is widely regarded as an adaptive regulation strategy (Gross and John, 2003). Within the process model of emotion regulation (Gross, 1998; Gross, 2015), cognitive reappraisal is classified as an antecedent-focused strategy that operates before full emotional responses unfold, distinguishing it from response-focused strategies such as expressive suppression and attentional deployment strategies such as distraction. Because psychological need frustration challenges students’ self-related appraisals and meaning-making processes, cognitive reappraisal is theoretically relevant for understanding how students respond to need-frustrating experiences.

The association between psychological need frustration and cognitive reappraisal may be complex rather than uniformly negative. On the one hand, need-frustrating experiences may be linked to psychological strain and reduced regulatory capacity. On the other hand, such experiences may also increase students’ perceived need to reinterpret stressful, self-threatening, or competence-related situations. From a compensatory regulation perspective, students who experience psychological need frustration may report greater use of cognitive reappraisal as an effortful attempt to make sense of pressure, perceived incompetence, or social difficulty. Cognitive reappraisal may therefore function not only as an indicator of regulatory capacity, but also as a coping-oriented response that becomes more salient under psychologically demanding conditions.

Individuals who frequently engage in cognitive reappraisal tend to report higher life satisfaction and more favorable affective outcomes (Gross and John, 2003). Accordingly, cognitive reappraisal may statistically account for part of the association between psychological need frustration and subjective well-being, although the cross-sectional design of the present study precludes conclusions about temporal or causal mediation.

*H2*: Psychological need frustration is associated with cognitive reappraisal.

*H3*: Cognitive reappraisal statistically accounts for part of the association between psychological need frustration and subjective well-being.

#### The moderating role of sport focus disruption

Although psychological need frustration may be associated with cognitive reappraisal, this association may vary according to the level of sport-related attentional disruption experienced by students in physical education contexts. Sport focus disruption refers to difficulties in maintaining task-relevant attention during performance situations (Smith et al., 2006). Drawing on Attentional Control Theory (Eysenck et al., 2007) and Self-Regulation Theory (Baumeister and Vohs, 2007), sport focus disruption can be understood as a condition under which performance pressure, self-evaluative concerns, and regulatory demands become more salient.

In physical education settings, students with higher sport focus disruption may be more aware of performance-related mistakes, competence threats, and evaluative pressure. Under such conditions, psychological need frustration may be more strongly associated with reported cognitive reappraisal because students may attempt to reinterpret need-frustrating experiences in order to maintain psychological adjustment. Rather than simply weakening regulatory capacity, sport focus disruption may amplify the salience of need-frustrating experiences and thereby make compensatory cognitive reappraisal more likely. From this perspective, sport focus disruption functions as a theoretically meaningful boundary condition that shapes the strength of the need frustration–reappraisal association.

In sport and physical education contexts, attentional disruption has been associated with elevated psychological pressure and reduced regulatory effectiveness (Parker et al., 2018). Although Parker et al. (2018) focused on athletes, the underlying principle that performance-related attentional demands can alter psychological processes may similarly apply to vocational students who face regular skill evaluations in physical education contexts.

*H4*: Sport focus disruption moderates the association between psychological need frustration and cognitive reappraisal, such that this association is stronger at higher levels of sport focus disruption.

*H5*: The indirect association between psychological need frustration and subjective well-being through cognitive reappraisal is conditional on levels of sport focus disruption.

## Methods

This study employed a cross-sectional survey design to examine a moderated mediation model in which psychological need frustration was associated with subjective well-being through cognitive reappraisal, with sport focus disruption examined as a moderator of the need frustration–reappraisal pathway. Given the cross-sectional nature of the data, the analytical model was interpreted as a pattern of conditional associations rather than as evidence of temporal or causal mediation.

### Participants

A multi-stage cluster sampling method was used to select participants from three vocational colleges in central China. Participants were drawn from five major fields of study: manufacturing, business services, information technology, healthcare, and tourism. All participants were enrolled in physical education courses as part of their regular curriculum. A total of 2,412 questionnaires were distributed, and 2,361 were returned, yielding a response rate of 97.9%. After excluding incomplete responses (defined as more than 20% missing items) and cases with straight-lining response patterns, the final valid sample consisted of 2,344 students (52% female, 48% male), aged 17 to 22 years (*M* = 19.2, *SD* = 1.3), with a valid response rate of 97.2%. The final sample size was considered adequate for testing the proposed moderated mediation model, given the large number of participants relative to the number of estimated parameters and prior studies using comparable models in educational and sport psychology contexts (Collie et al., 2019; Parker et al., 2018).

### Measures

#### Instrument adaptation for the present sample

Existing Chinese versions of the instruments were used where available, and all items were reviewed for clarity and contextual relevance in the physical education setting. For the Sport Anxiety Scale-2 Focus Disruption subscale, the English version was translated into Chinese by two bilingual researchers working independently. The two forward translations were synthesized through discussion, and the synthesized version was back-translated into English by a third bilingual researcher blind to the original instrument. Discrepancies were resolved through consensus. Prior to the main study, the complete questionnaire was piloted with 45 vocational college students (not included in the final sample) to evaluate item clarity and response time. Minor wording adjustments were made based on pilot feedback. Table 1 summarizes the psychometric properties of all instruments in the present sample.

**Table 1:** Internal consistency of the study measures in the present sample.

Measure	Items	Cronbach’s *α*	McDonald’s *ω*
Psychological need frustration	9	0.932	0.933
Cognitive reappraisal	6	0.939	0.939
Sport focus disruption	5	0.890	0.892
Subjective well-being, 3-item SHS	3	0.935	0.935
Subjective well-being, 4-item SHS	4	0.685	0.819

#### Psychological need frustration

Psychological need frustration was operationalized as a derived indicator using the nine negatively worded items from the Chinese version of the Basic Needs Satisfaction in General Scale (BNSG-S; Gagné, 2003; Liu et al., 2013). The BNSG-S assesses autonomy, competence, and relatedness need experiences using a 7-point Likert scale (1 = strongly disagree, 7 = strongly agree). In the original scoring procedure, negatively worded items are reverse-coded so that higher scores indicate greater basic psychological need satisfaction. Because the present study focused on psychological need frustration, these nine items were re-transformed so that higher scores represented greater psychological need frustration. The derived psychological need frustration score demonstrated excellent internal consistency in the present sample (Cronbach’s *α* = 0.932, McDonald’s *ω* = 0.933).

#### Cognitive reappraisal

Cognitive reappraisal was measured using the Cognitive Reappraisal subscale of the Emotion Regulation Questionnaire (ERQ; Gross and John, 2003). The subscale contains 6 items rated on a 7-point Likert scale (1 = strongly disagree, 7 = strongly agree). A sample item is: “When I want to feel less negative emotion, I change the way I’m thinking about the situation.” Cognitive reappraisal showed excellent internal consistency in the present sample (*α* = 0.939, *ω* = 0.939).

#### Sport focus disruption

Sport focus disruption was assessed using the Focus Disruption subscale of the Sport Anxiety Scale-2 (SAS-2; Smith et al., 2006). The subscale includes 5 items rated on a 4-point Likert scale (1 = not at all, 4 = very much). A sample item is: “During physical activities, I have difficulty concentrating on what I am supposed to do.” Sport focus disruption showed good internal consistency in the present sample (*α* = 0.890, *ω* = 0.892).

#### Subjective well-being

Subjective well-being was measured using the Subjective Happiness Scale (SHS; Lyubomirsky and Lepper, 1999). The original scale contains four items rated on a 7-point scale, including one reverse-coded item. In the present sample, the original four-item scale showed lower internal consistency (Cronbach’s *α* = 0.685, McDonald’s *ω* = 0.819), whereas the three positively worded items demonstrated excellent internal consistency (Cronbach’s *α* = 0.935, McDonald’s *ω* = 0.935). Therefore, the three-item SHS score was used as the primary subjective well-being measure, and the original four-item score was retained for sensitivity analyses. This issue is further acknowledged in the Limitations section.

#### Confirmatory factor analysis

A confirmatory factor analysis was conducted to evaluate the measurement structure of the main study variables. The four-factor model, including psychological need frustration, cognitive reappraisal, sport focus disruption, and subjective well-being as correlated latent factors, showed acceptable fit, *χ*^2^(224) = 3063.51, *p* < 0.001, CFI = 0.932, TLI = 0.923, RMSEA = 0.074, 90% *CI* [0.071, 0.076]. Standardized factor loadings ranged from 0.676 to 0.912.

#### Data collection procedure

Data were collected between September and October 2025. The study was approved by the Office of Academic Research, Nanyang Vocational College of Agriculture (Approval No. UCS-2025072207, July 22, 2025). Written informed consent was obtained from all adult participants and from the parents or legal guardians of participants under 18 years of age prior to survey administration. Questionnaires were administered in a paper-and-pencil format during regular class sessions under the supervision of trained research assistants. To reduce potential common method bias, several procedural safeguards were implemented: (a) responses were collected anonymously; (b) the order of scales was counterbalanced across questionnaire versions; and (c) where applicable, both positively and negatively worded items were retained in the questionnaire to reduce response-pattern tendencies.

### Statistical analysis

All analyses were conducted using SPSS 27.0. Prior to hypothesis testing, data were screened for missing values, univariate and multivariate outliers, and violations of normality. Descriptive statistics and bivariate correlations were computed for all study variables.

### Common method bias

As noted above, procedural safeguards were implemented during data collection, including anonymous responding, counterbalanced scale order, and the inclusion of both positively and negatively worded items where applicable. In addition, Harman’s single-factor test was conducted to evaluate the potential influence of common method variance. Exploratory factor analysis of all scale items using an unrotated solution yielded a first factor accounting for 28.3% of the total variance, below the commonly referenced 40% threshold (Podsakoff et al., 2003). These results suggested that common method variance was unlikely to fully account for the observed associations. Nevertheless, because all core variables were assessed via self-report, common method bias cannot be completely ruled out, and this is acknowledged as a limitation.

### Moderated mediation analysis

Moderated mediation analyses were conducted using the PROCESS macro (Version 4.2; Hayes, 2022) for SPSS, employing Model 7. This model estimates the conditional indirect effect of an independent variable on a dependent variable through a mediator, with the path between the independent variable and mediator moderated by a fourth variable. In the present analysis, psychological need frustration served as the independent variable (X), cognitive reappraisal as the mediator (M), sport focus disruption as the moderator (W), and subjective well-being (3-item SHS) as the dependent variable (Y). Age, gender, and weekly exercise time were included as covariates. Continuous predictors were mean-centered before creating the interaction term. Percentile bootstrap confidence intervals (95% *CI*) based on 5,000 resamples were used to evaluate the indirect and conditional indirect effects. An effect was considered statistically significant when the 95% *CI* did not include zero. Consistent with the cross-sectional design, all reported associations represent conditional patterns in the observed data rather than causal or temporal mediation. Sensitivity analyses were conducted using the original 4-item SHS score to evaluate the robustness of the findings.

### Control variables

Age and gender were included as covariates because these demographic factors are commonly associated with subjective well-being and emotion regulation strategy use in young adult populations (Nolen-Hoeksema and Aldao, 2011; Diener et al., 2018). Weekly exercise time was assessed using a four-category item asking participants to report their average weekly time spent in physical activity (<1 h, 1–2 h, 3–4 h, or ≥5 h), and was included as an additional covariate given the physical education context of the study and prior evidence linking physical activity to both well-being and emotion regulation. This approach was adopted to preserve a theoretically focused model while accounting for key demographic and contextual variables. Other potentially relevant covariates—including socioeconomic status, academic performance, and baseline mental health—were not consistently available across participants and were therefore not included. We recognize that omitted variables may contribute to residual confounding, and this is addressed in the Limitations section.

## Results

### Data quality and preliminary analysis

A total of 2,344 valid questionnaires were collected from students enrolled in three vocational colleges in China. Participants ranged in age from 17 to 22 years (*M* = 19.2, *SD* = 1.3), with 52.1% female and 47.9% male students. Participants were drawn from five major fields of study: manufacturing, business services, information technology, healthcare, and tourism. Descriptive statistics for the core study variables are presented in Table 2.

**Table 2:** Descriptive statistics of the core study variables.

Variable	*M*	SD
Psychological need frustration	3.41	1.17
Sport focus disruption	1.58	0.55
Cognitive reappraisal	4.72	1.28
Subjective well-being	4.55	1.35

*M* = mean; SD = standard deviation.

A four-factor confirmatory factor analysis was conducted to evaluate the measurement structure of the main study variables. The model included psychological need frustration, cognitive reappraisal, sport focus disruption, and subjective well-being as correlated latent factors. The model showed acceptable fit, *χ*^2^(224) = 3063.51, *p* < 0.001, CFI = 0.932, TLI = 0.923, RMSEA = 0.074, 90% *CI* [0.071, 0.076]. Standardized factor loadings ranged from 0.676 to 0.912.

To assess potential common method bias, Harman’s single-factor test was conducted. The first unrotated factor accounted for 28.3% of the total variance, which was below the commonly referenced 40% threshold. This result suggested that common method variance was unlikely to fully account for the observed associations. Nevertheless, given the self-report and cross-sectional design, common method bias cannot be completely ruled out.

### Correlation analysis

Pearson correlation analyses were conducted to examine associations among psychological need frustration, sport focus disruption, cognitive reappraisal, and subjective well-being (see Table 3). Psychological need frustration was negatively correlated with subjective well-being measured using the three-item SHS composite (*r* = −0.119, *p* < 0.001) and positively correlated with sport focus disruption (*r* = 0.439, *p* < 0.001), but it was not significantly correlated with cognitive reappraisal at the zero-order level (*r* = −0.002, *p* = 0.937). Cognitive reappraisal was positively correlated with subjective well-being (*r* = 0.529, *p* < 0.001). Sport focus disruption was negatively correlated with subjective well-being (*r* = −0.207, *p* < 0.001) and cognitive reappraisal (*r* = −0.133, *p* < 0.001). Variance inflation factor values were below 5, indicating no substantial multicollinearity concerns.

**Table 3:** Means, standard deviations, and correlations among study variables.

Variable	*M*	SD	1	2	3	4
Psychological need frustration	3.41	1.17	–			
Sport focus disruption	1.58	0.55	0.439**	–		
Cognitive reappraisal	4.72	1.28	−0.002	−0.133**	–	
Subjective well-being	4.55	1.35	−0.119**	−0.207**	0.529**	–

M = mean; SD = standard deviation. **p < 0.01.

### Moderated mediation analysis

A moderated mediation analysis was conducted using PROCESS Model 7 with 5,000 bootstrap samples. Psychological need frustration was entered as the independent variable, cognitive reappraisal as the mediator, sport focus disruption as the moderator, and subjective well-being measured by the three-item SHS composite as the dependent variable. Age, gender, and weekly exercise time were included as covariates. Continuous predictors were mean-centered before creating the interaction term.

In the model predicting cognitive reappraisal, the overall model was significant, *R* = 0.2165, *R*^2^ = 0.0469, *F*(6, 2,337) = 19.1547, *p* < 0.001. Psychological need frustration was positively associated with cognitive reappraisal (*b* = 0.1249, *SE* = 0.0254, *t* = 4.9202, *p* < 0.001, 95% *CI* [0.0751, 0.1747]). Sport focus disruption was negatively associated with cognitive reappraisal (*b* = −0.5078, *SE* = 0.0570, *t* = −8.9160, *p* < 0.001, 95% *CI* [−0.6195, −0.3961]). The interaction between psychological need frustration and sport focus disruption was significant (*b* = 0.2302, *SE* = 0.0325, *t* = 7.0785, *p* < 0.001, 95% *CI* [0.1664, 0.2939]), indicating that sport focus disruption moderated the association between psychological need frustration and cognitive reappraisal.

Conditional effect analyses showed that the association between psychological need frustration and cognitive reappraisal was not significant at low levels of sport focus disruption (−1 *SD*: *b* = −0.0028, *SE* = 0.0274, *t* = −0.1019, *p* = 0.9189, 95% *CI* [−0.0566, 0.0510]). However, this association was significant and positive at mean levels of sport focus disruption (*b* = 0.1249, *SE* = 0.0254, *t* = 4.9202, *p* < 0.001, 95% *CI* [0.0751, 0.1747]) and became stronger at high levels of sport focus disruption (+1 *SD*: *b* = 0.2526, *SE* = 0.0344, *t* = 7.3334, *p* < 0.001, 95% *CI* [0.1851, 0.3202]). The Johnson–Neyman analysis indicated that the association between psychological need frustration and cognitive reappraisal became statistically significant when sport focus disruption exceeded −0.3288.

In the model predicting subjective well-being, the overall model was significant, *R* = 0.5534, *R*^2^ = 0.3062, *F*(5, 2,338) = 206.3990, *p* < 0.001. Psychological need frustration was negatively associated with subjective well-being (*b* = −0.1186, *SE* = 0.0201, *t* = −5.8913, *p* < 0.001, 95% *CI* [−0.1581, −0.0791]). Cognitive reappraisal was positively associated with subjective well-being (*b* = 0.5492, *SE* = 0.0183, *t* = 30.0542, *p* < 0.001, 95% *CI* [0.5134, 0.5850]). Weekly exercise time was also positively associated with subjective well-being (*b* = 0.1588, *SE* = 0.0270, *t* = 5.8838, *p* < 0.001, 95% *CI* [0.1059, 0.2117]). Age and gender were not significant predictors in this model.

The conditional indirect association between psychological need frustration and subjective well-being through cognitive reappraisal varied by levels of sport focus disruption (Table 4). At low levels of sport focus disruption, the indirect association was not significant (effect = −0.0015, *BootSE* = 0.0223, 95% *CI* [−0.0453, 0.0423]). At mean levels of sport focus disruption, the indirect association was significant and positive (effect = 0.0686, *BootSE* = 0.0194, 95% *CI* [0.0294, 0.1065]). At high levels of sport focus disruption, the indirect association was also significant and stronger in magnitude (effect = 0.1387, *BootSE* = 0.0234, 95% *CI* [0.0902, 0.182]). The index of moderated mediation was significant (index = 0.1264, *BootSE* = 0.0218, 95% *CI* [0.0792, 0.1655]), indicating that the indirect association through cognitive reappraisal was conditional on sport focus disruption.

**Table 4:** Conditional indirect effects of psychological need frustration on subjective well-being at different levels of sport focus disruption.

Sport focus disruption level	Indirect effect	BootSE	95% CI
Low (−1 SD)	−0.0015	0.0223	[−0.0453, 0.0423]
Mean	0.0686	0.0194	[0.0294, 0.1065]
High (+1 SD)	0.1387	0.0234	[0.0902, 0.1820]

BootSE, bootstrap standard error; CI, confidence interval based on 5,000 bootstrap samples. The dependent variable was subjective well-being measured using the three-item SHS composite.

### Sensitivity analysis using the four-item SHS

Sensitivity analyses were conducted using the original four-item SHS score as the dependent variable. The results were substantively similar to the main analysis. Psychological need frustration remained negatively associated with subjective well-being (*b* = −0.2735, *SE* = 0.0151, *t* = −18.1659, *p* < 0.001, 95% *CI* [−0.3030, −0.2440]), and cognitive reappraisal remained positively associated with subjective well-being (*b* = 0.3843, *SE* = 0.0137, *t* = 28.1222, *p* < 0.001, 95% *CI* [0.3575, 0.4111]). The conditional indirect association was not significant at low levels of sport focus disruption (effect = −0.0011, *BootSE* = 0.0156, 95% *CI* [−0.0309, 0.0297]), but was significant at mean levels (effect = 0.0480, *BootSE* = 0.0138, 95% *CI* [0.0212, 0.0751]) and high levels of sport focus disruption (effect = 0.0971, *BootSE* = 0.0170, 95% *CI* [0.0622, 0.1296]). The index of moderated mediation remained significant (index = 0.0885, *BootSE* = 0.0156, 95% *CI* [0.0557, 0.1172]). These findings indicate that the main moderated mediation pattern was robust when subjective well-being was measured using the original four-item SHS.

## Discussion

The present study examined associations between psychological need frustration, cognitive reappraisal, sport focus disruption, and subjective well-being among vocational college students in physical education contexts. Three main findings emerged. First, psychological need frustration was negatively associated with subjective well-being, supporting the relevance of Self-Determination Theory for understanding well-being in vocational physical education settings. Second, cognitive reappraisal was positively associated with subjective well-being and statistically accounted for part of the association between psychological need frustration and subjective well-being. Third, sport focus disruption moderated the association between psychological need frustration and cognitive reappraisal. Specifically, psychological need frustration was not significantly associated with cognitive reappraisal at low levels of sport focus disruption, but it was positively associated with cognitive reappraisal at average and high levels of sport focus disruption. The conditional indirect association through cognitive reappraisal was also significant at average and high levels of sport focus disruption, but not at low levels.

These findings provide a more nuanced picture than a simple risk pathway in which need frustration uniformly undermines adaptive emotion regulation. Consistent with Self-Determination Theory, students who reported greater psychological need frustration tended to report lower subjective well-being. However, the pattern involving cognitive reappraisal suggests that need-frustrating experiences may also be associated with greater reported use of reappraisal under certain conditions. This finding is consistent with a compensatory regulation interpretation: when students experience both need-related strain and attentional disruption in physical education settings, they may be more likely to reinterpret stressful or self-threatening experiences in an effort to maintain psychological adjustment. In this sense, cognitive reappraisal may not only reflect regulatory capacity, but may also function as a coping-oriented response that becomes more salient when students encounter psychologically demanding situations.

The moderating role of sport focus disruption is particularly important. Sport focus disruption was negatively associated with cognitive reappraisal at the main-effect level, suggesting that attentional disruption may generally coincide with lower use of adaptive regulation. Yet the significant interaction indicated that the association between psychological need frustration and cognitive reappraisal became stronger as sport focus disruption increased. This pattern suggests that sport focus disruption may operate as a boundary condition under which need-frustrating experiences become more psychologically salient. In performance-oriented physical education contexts, students who experience difficulty maintaining focus may also become more aware of mistakes, competence concerns, peer comparison, and evaluative pressure. Under these conditions, psychological need frustration may be more strongly linked to attempts to reinterpret the situation, possibly as a compensatory response to heightened psychological demand.

The conditional indirect effects further support this interpretation. At low levels of sport focus disruption, psychological need frustration was not indirectly associated with subjective well-being through cognitive reappraisal. At average and high levels of sport focus disruption, however, the indirect association was significant and positive. Although the conditional indirect effects were statistically significant at average and high levels of sport focus disruption, their absolute magnitudes were modest. Therefore, these effects should be interpreted as indicating a conditional regulatory pattern rather than a large practical effect. This positive indirect association should be interpreted alongside the negative direct association between psychological need frustration and subjective well-being. Taken together, the results suggest a mixed pattern: psychological need frustration was directly associated with lower subjective well-being, but under higher sport focus disruption it was also associated with greater cognitive reappraisal, which in turn was associated with higher subjective well-being. This pattern may reflect a compensatory psychological process rather than a straightforward harmful pathway. Because the data were cross-sectional, however, this interpretation remains tentative and should not be read as evidence that need frustration causes greater reappraisal or that reappraisal causally improves well-being.

### Implications for physical education practice

Although the findings are associational, they offer several preliminary implications for physical education practice in vocational colleges. Physical education classes often involve skill demonstration, peer comparison, performance evaluation, and immediate feedback. These features may make competence concerns and attentional disruption especially salient for students who already experience psychological need frustration. Instructors may therefore consider strategies that reduce unnecessary competence threat and support students’ regulatory engagement. Examples include emphasizing self-referenced progress rather than peer comparison, providing autonomy-supportive feedback, normalizing mistakes during skill acquisition, and using brief task-focused cues to help students refocus attention during performance activities.

The finding that cognitive reappraisal was positively associated with subjective well-being also suggests that emotion regulation support may be relevant in physical education settings. For example, teachers could encourage students to reinterpret temporary mistakes as part of skill development rather than as indicators of low ability. However, such implications should be treated as hypothesis-generating rather than prescriptive. The present data do not establish whether these instructional strategies would increase reappraisal, reduce need frustration, or improve well-being. Intervention-based and longitudinal studies are needed before stronger practice recommendations can be made.

### Cultural and educational context

The findings should also be interpreted in light of the Chinese vocational education context. Vocational college students may experience distinctive pressures related to employment uncertainty, perceived educational status, and skill-based evaluation. These contextual features may shape how students interpret competence-related challenges and regulate emotional responses during physical education. The present results suggest that vocational physical education settings provide a meaningful context for examining how motivational strain, attentional disruption, and emotion regulation are jointly associated with well-being. Future research should test whether similar patterns are observed in general universities, different vocational systems, and other cultural contexts.

### Limitations and future directions

Several limitations should be noted. First, the cross-sectional design prevents conclusions about temporal ordering or causal mediation. Although the model was theoretically specified, the observed paths should be interpreted as conditional associations. Longitudinal and experimental designs are needed to examine whether psychological need frustration precedes changes in cognitive reappraisal and subjective well-being over time. Second, all core variables were assessed through self-report questionnaires, which may introduce shared method variance and response biases. Although procedural safeguards and Harman’s single-factor test suggested that common method variance was unlikely to fully account for the findings, it cannot be ruled out. Future studies could further evaluate common method variance using marker-variable approaches or multi-source data (Lindell and Whitney, 2001). Third, subjective well-being was primarily assessed using the three positively worded SHS items because the original four-item scale showed lower internal consistency in this sample. Although sensitivity analyses using the original four-item SHS produced substantively similar results, future studies should further evaluate the performance of the reverse-coded SHS item in vocational student samples.

Fourth, psychological need frustration was operationalized as a derived indicator based on negatively worded items from the BNSG-S. Although this approach was consistent with the present study’s focus and demonstrated strong internal consistency, future research would benefit from using instruments specifically designed to distinguish need satisfaction and need frustration as separate constructs, such as the Basic Psychological Need Satisfaction and Frustration Scale (Chen et al., 2015). Finally, the present study did not include objective indicators of physical education performance, attentional control, injury risk, or physiological fatigue. Future studies could incorporate behavioral or physiological measures to examine whether the psychological patterns observed here are linked to functional outcomes in physical education and sport-related settings.

Overall, this study extends research on vocational college students’ subjective well-being by integrating Self-Determination Theory, emotion regulation theory, and attentional control perspectives. The findings suggest that psychological need frustration is associated with lower subjective well-being, while cognitive reappraisal may represent a compensatory regulatory pathway under conditions of higher sport focus disruption. By emphasizing conditional patterns rather than causal claims, the study offers a more cautious account of how motivational strain, emotion regulation, and sport-related attentional disruption are jointly associated with well-being in vocational physical education contexts.

## Conclusion

This study examined associations among psychological need frustration, cognitive reappraisal, sport focus disruption, and subjective well-being in a large sample of Chinese vocational college students in physical education contexts. Psychological need frustration was negatively associated with subjective well-being, supporting the relevance of need-related experiences for students’ psychological adjustment. Cognitive reappraisal was positively associated with subjective well-being and statistically accounted for part of the association between psychological need frustration and subjective well-being under specific conditions.

Sport focus disruption functioned as a contextual boundary condition. The association between psychological need frustration and cognitive reappraisal was not significant at low levels of sport focus disruption, but became positive and stronger at average and high levels of sport focus disruption. Similarly, the indirect association between psychological need frustration and subjective well-being through cognitive reappraisal was significant only at average and high levels of sport focus disruption. This pattern suggests that cognitive reappraisal may reflect a compensatory regulatory response when students experience both need-related strain and attentional disruption.

These findings contribute to understanding how motivational, regulatory, and attentional processes are jointly associated with subjective well-being in vocational physical education settings. Rather than identifying a simple risk pathway, the results point to a conditional pattern in which psychological need frustration is directly linked to lower subjective well-being, while cognitive reappraisal may serve as a potentially adaptive regulatory process under higher sport focus disruption. Given the cross-sectional design, all findings should be interpreted as associations rather than evidence of causal or temporal mediation. Future longitudinal and experimental studies are needed to clarify the directionality and practical implications of these conditional associations.

## Practical implications

The findings offer preliminary implications for vocational education and physical education practice. Students who experience psychological need frustration and sport focus disruption may face heightened regulatory demands during physical education activities. Instructors may therefore consider creating learning environments that reduce unnecessary competence threat and excessive social comparison, provide autonomy-supportive feedback, and normalize mistakes as part of skill development. Brief attentional refocusing strategies and guided cognitive reappraisal exercises may also help students reinterpret performance difficulties in more adaptive ways.

These implications should be treated cautiously. The present findings do not demonstrate that such strategies will directly improve cognitive reappraisal or subjective well-being. Instead, they suggest that physical education settings may be a useful context for supporting students’ motivational needs, attentional focus, and emotion regulation. Intervention-based studies are needed to test whether these approaches can improve students’ well-being and learning experiences.

## Data Availability

The authors will make the de-identified raw data supporting the conclusions of this article available without undue reservation.
